# Co-infection and ICU-acquired infection in COIVD-19 ICU patients: a secondary analysis of the UNITE-COVID data set

**DOI:** 10.1186/s13054-022-04108-8

**Published:** 2022-08-03

**Authors:** Andrew Conway Morris, Katharina Kohler, Thomas De Corte, Ari Ercole, Harm-Jan De Grooth, Paul W. G. Elbers, Pedro Povoa, Rui Morais, Despoina Koulenti, Sameer Jog, Nathan Nielsen, Alasdair Jubb, Maurizio Cecconi, Jan De Waele, for the ESICM UNITE COVID investigators, Marco Bezzi, Alicia Gira, Philipp Eller, Tarikul Hamid, Injamam Ull Haque, Wim De Buyser, Antonella Cudia, Daniel De Backer, Pierre Foulon, Vincent Collin, Jan De Waele, Jolien Van Hecke, Elisabeth De Waele, Claire Van Malderen, Jean-Baptiste Mesland, Michael Piagnerelli, Lionel Haentjens, Nicolas De Schryver, Jan Van Leemput, Philippe Vanhove, Pierre Bulpa, Viktoria Ilieva, David Katz, Anna Geagea, Alexandra Binnie, Fernando Tirapegui, Gustavo Lago, Jerónimo Graf, Rodrigo Perez-Araos, Patricio Vargas, Felipe Martinez, Eduardo Labarca, Daniel Molano Franco, Daniela Parra-Tanoux, Luis Felipe Reyes, David Yepes, Filip Periš, Sanda Stojanović Stipić, Cynthia Vanessa Campozano Burgos, Paulo Roberto Navas Boada, Jose Luis Barberan Brun, Juan Pablo Paredes Ballesteros, Ahmed Hammouda, Omar Elmandouh, Ahmed Azzam, Aliae Mohamed Hussein, Islam Galal, Ahmed K. Awad, Mohammed A. Azab, Maged Abdalla, Hebatallah Assal, Mostafa Alfishawy, Sherief Ghozy, Samar Tharwat, Abdullah Eldaly, Veronika Reinhard, Anne Chrisment, Chrystelle Poyat, Julio Badie, Fernando Berdaguer Ferrari, Björn Weiss, Karl Friedrich Kuhn, Julius J. Grunow, Marco Lorenz, Stefan Schaller, Peter Spieth, Marc Bota, Falk Fichtner, Kristina Fuest, Tobias Lahmer, Johannes Herrmann, Patrick Meybohm, Nikolaos Markou, Georgia Vasileiadou, Evangelia Chrysanthopoulou, Panagiotis Papamichalis, Ioanna Soultati, Sameer Jog, Kushal Kalvit, Sheila Nainan Myatra, Ivan Krupa, Aisa Tharwat, Alistair Nichol, Aine McCarthy, Ata Mahmoodpoor, Tommaso Tonetti, Paolo Isoni, Savino Spadaro, Carlo Alberto Volta, Lucia Mirabella, Alberto Noto, Gaetano Florio, Amedeo Guzzardella, Chiara Paleari, Federica Baccanelli, Marzia Savi, Massimo Antonelli, Barbara Vaccarini, Giorgia Montrucchio, Gabriele Sales, Katia Donadello, Leonardo Gottin, Enrico Polati, Silvia De Rosa, Demet Sulemanji, Abdurraouf Abusalama, Muhammed Elhadi, Montelongo Felipe De Jesus, Daniel Rodriguez Gonzalez, Nancy Canedo, Alejandro Esquivel Chavez, Tarek Dendane, Bart Grady, Ben de Jong, Eveline van der Heiden, Patrick Thoral, Bas van den Bogaard, Peter E. Spronk, Sefanja Achterberg, Melanie Groeneveld, Ralph K. L. So, Calvin de Wijs, Harm Scholten, Albertus Beishuizen, Alexander D. Cornet, Auke C. Reidinga, Hetty Kranen, Roos Mensink, Sylvia den Boer, Marcel de Groot, Oliver Beck, Carina Bethlehem, Bas van Bussel, Tim Frenzel, Celestine de Jong, Rob Wilting, Jozef Kesecioglu, Jannet Mehagnoul-Schipper, Datonye Alasia, Ashok Kumar, Ahad Qayyum, Muhammad Rana, Mustafa Abu Jayyab, Rosario Quispe Sierra, Aaron Mark Hernandez, Lúcia Taborda, Tiago Ramires, Catarina Silva, Carolina Roriz, Pedro Póvoa, Patricia Patricio, Maria Lurdes Santos, Vasco Costa, Pedro Cunha, Celina Gonçalves, Sandra Nunes, João Camões, Diana Adrião, Ana Oliveira, Ali Omrani, Muna Al Maslamani, Abdurrahmaan Suei elbuzidi, Bara Mahmoud Al qudah, Abdel Rauof Akkari, Mohamed Alkhatteb, Anas Baiou, Ahmed Husain, Mohamed Alwraidat, Ibrahim Abdulsalam Saif, Dana Bakdach, Amna Ahmed, Mohamed Aleef, Awadh Bintaher, Cristina Petrisor, Evgeniy Popov, Ksenia Popova, Mariia Dementienko, Boris Teplykh, Alexey Pyregov, Liubov Davydova, Belskii Vladislav, Elena Neporada, Ivan Zverev, Svetlana Meshchaninova, Dmitry Sokolov, Elena Gavrilova, Irena Shlyk, Igor Poliakov, Mapинa Bлacoвa, Ohoud Aljuhani, Amina Alkhalaf, Felwa Bin Humaid, Yaseen Arabi, Ahmed Kuhail, Omar Elrabi, Madihah Alghnam, Amit Kansal, Vui Kian Ho, Jensen Ng, Raquel Rodrígez García, Xiana Taboada Fraga, Mª del Pilar García-Bonillo, Antonio Padilla-Serrano, Marta Martin Cuadrado, Carlos Ferrando, Ignacio Catalan-Monzon, Laura Galarza, Fernando Frutos-Vivar, Jorge Jimenez, Carmen Rodríguez-Solis, Enric Franquesa-Gonzalez, Guillermo Pérez Acosta, Luciano Santana Cabrera, Juan Pablo Aviles Parra, Francisco Muñoyerro Gonzalez, Maria del Carmen Lorente Conesa, Ignacio Yago Martinez Varela, Orville Victoriano Baez Pravia, Maria Cruz Martin Delgado, Carlos Munoz de Cabo, Ana-Maria Ioan, Cesar Perez-Calvo, Arnoldo Santos, Ane Abad-Motos, Javier Ripolles-Melchor, Belén Civantos Martin, Santiago Yus Teruel, Juan Higuera Lucas, Aaron Blandino Ortiz, Raúl de Pablo Sánchez, Jesús Emilio Barrueco-Francioni, Lorena Forcelledo Espina, José M. Bonell-Goytisolo, Iñigo Salaverria, Antonia Socias Mir, Emilio Rodriguez-Ruiz, Virginia Hidalgo Valverde, Patricia Jimeno Cubero, Francisca Arbol Linde, Nieves Cruza Leganes, Juan Maria Romeu, Pablo Concha, José Angel Berezo-Garcia, Virginia Fraile, Cristina Cuenca-Rubio, David Perez-Torres, Ainhoa Serrano, Clara Martínez Valero, Andrea Ortiz Suner, Leire Larrañaga, Noemi Legaristi, Gerardo Ferrigno, Safa Khlafalla, Rosita Bihariesingh-Sanchit, Frank Zoerner, Jonathan Grip, Kristina Kilsand, Jonas Österlind, Magnus von Seth, Johan Berkius, Samuele Ceruti, Andrea Glotta, Seval Izdes, Işıl Özkoçak Turan, Ahmet Cosar, Burcin Halacli, Necla Dereli, Mehmet Yilmaz, Türkay Akbas, Gülseren Elay, Selin Eyüpoğlu, Yelíz Bílír, Kemal Tolga Saraçoğlu, Ebru Kaya, Ayca Sultan Sahin, Pervin Korkmaz Ekren, Tuğçe Mengi, Kezban Ozmen Suner, Yakup Tomak, Ahmet Eroglu, Asad Alsabbah, Katie Hanlon, Kevin Gervin, Sean McMahon, Samantha Hagan, Caroline V. Higenbottam, Randeep Mullhi, Lottie Poulton, Tomasz Torlinski, Allen Gareth, Nick Truman, Gopal Vijayakumar, Chris Hall, Alasdair Jubb, Lenka Cagova, Nicola Jones, Sam Graham, Nicole Robin, Amanda Cowton, Adrian Donnelly, Natalia Singatullina, Melanie Kent, Carole Boulanger, Zoë Campbell, Elizabeth Potter, Natalie Duric, Tamas Szakmany, Orinta Kviatkovske, Nandor Marczin, Caroline Ellis, Rajnish Saha, Chunda Sri-Chandana, John Allan, Lana Mumelj, Harish Venkatesh, Vera Nina Gotz, Anthony Cochrane, Nuttha Lumlertgul, Barbara Ficial, Susan Jain, Giulia Beatrice Crapelli, Aikaterini Vlachou, David Golden, Sweyn Garrioch, Jeremy Henning, Gupta Loveleena, Miriam Davey, Lina Grauslyte, Erika Salciute-Simene, Martin Cook, Danny Barling, Phil Broadhurst, Sarah Purvis, Spivey Michael, Benjamin Shuker, Irina Grecu, Daniel Harding, Natalia Singatullina, James T. Dean, Nathan D. Nielsen, Sama Al-Bayati, Mohammed Al-Sadawi, Mariane Charron, Peter Stubenrauch, Jairo Santanilla, Catherine Wentowski, Dorothea Rosenberger, Polikseni Eksarko, Randeep Jawa

**Affiliations:** 1 Division of Anaesthesia, Department of Medicine, Level 4 Addenbrooke’s Hospital, University of Cambridge, Hills Road, Cambridge, UK; 2 Division of Immunology, Department of Pathology, University of Cambridge, Cambridge, UK; 3 JVF Intensive Care Unit, Addenbrooke’s Hospital, Cambridge, UK; 4 Department of Intensive Care Medicine, Ghent University Hospital, Ghent, Belgium; 5 Dept of Internal Medicine and Pediatrics, Faculty of Medicine and Health Sciences, Ghent University, Ghent, Belgium; 6 Neurocritical Care Unit, Addenbrooke’s Hospital, Cambridge, UK; 7 Department of Intensive Care, Amsterdam UMC Location Vrije Universiteit Amsterdam, Amsterdam, The Netherlands; 8 Laboratory for Critical Care Computational Intelligence, Amsterdam Medical Data Science, Amsterdam UMC, Vrije Universiteit, Amsterdam, The Netherlands; 9 Nova Medical School, New University, Lisbon, Portugal; 10 Center for Clinical Epidemiology and Research Unit of Clinical Epidemiology, OUH Odense University Hospital, Odense, Denmark; 11 Polyvalent Intensive Care Unit, Hospital de São Francisco Xavier, CHLO, Lisbon, Portugal; 12 2Nd Critical Care Department, Attikon University Hospital, University of Athens, Athens, Greece; 13 UQ Centre for Clinical Research (UQCCR), Faculty of Medicine, The University of Queensland, Brisbane, Australia; 14 Deenanath Mangeshkar Hospital and Research Center, Pune, India; 15 Divisions of Pulmonary, Critical Care and Sleep Medicine and Transfusion Medicine, University of New Mexico School of Medicine, Albuquerque, NM USA; 16 Department of Anaesthesia, Humanitas University, Milan, Italy

## Abstract

**Background:**

The COVID-19 pandemic presented major challenges for critical care facilities worldwide. Infections which develop alongside or subsequent to viral pneumonitis are a challenge under sporadic and pandemic conditions; however, data have suggested that patterns of these differ between COVID-19 and other viral pneumonitides. This secondary analysis aimed to explore patterns of co-infection and intensive care unit-acquired infections (ICU-AI) and the relationship to use of corticosteroids in a large, international cohort of critically ill COVID-19 patients.

**Methods:**

This is a multicenter, international, observational study, including adult patients with PCR-confirmed COVID-19 diagnosis admitted to ICUs at the peak of wave one of COVID-19 (February 15th to May 15th, 2020). Data collected included investigator-assessed co-infection at ICU admission, infection acquired in ICU, infection with multi-drug resistant organisms (MDRO) and antibiotic use. Frequencies were compared by Pearson’s Chi-squared and continuous variables by Mann–Whitney U test. Propensity score matching for variables associated with ICU-acquired infection was undertaken using R library MatchIT using the “full” matching method.

**Results:**

Data were available from 4994 patients. Bacterial co-infection at admission was detected in 716 patients (14%), whilst 85% of patients received antibiotics at that stage. ICU-AI developed in 2715 (54%). The most common ICU-AI was bacterial pneumonia (44% of infections), whilst 9% of patients developed fungal pneumonia; 25% of infections involved MDRO. Patients developing infections in ICU had greater antimicrobial exposure than those without such infections. Incident density (ICU-AI per 1000 ICU days) was in considerable excess of reports from pre-pandemic surveillance. Corticosteroid use was heterogenous between ICUs. In univariate analysis, 58% of patients receiving corticosteroids and 43% of those not receiving steroids developed ICU-AI. Adjusting for potential confounders in the propensity-matched cohort, 71% of patients receiving corticosteroids developed ICU-AI vs 52% of those not receiving corticosteroids. Duration of corticosteroid therapy was also associated with development of ICU-AI and infection with an MDRO.

**Conclusions:**

In patients with severe COVID-19 in the first wave, co-infection at admission to ICU was relatively rare but antibiotic use was in substantial excess to that indication. ICU-AI were common and were significantly associated with use of corticosteroids.

*Trial registration* ClinicalTrials.gov: NCT04836065 (retrospectively registered April 8th 2021).

**Graphical abstract:**

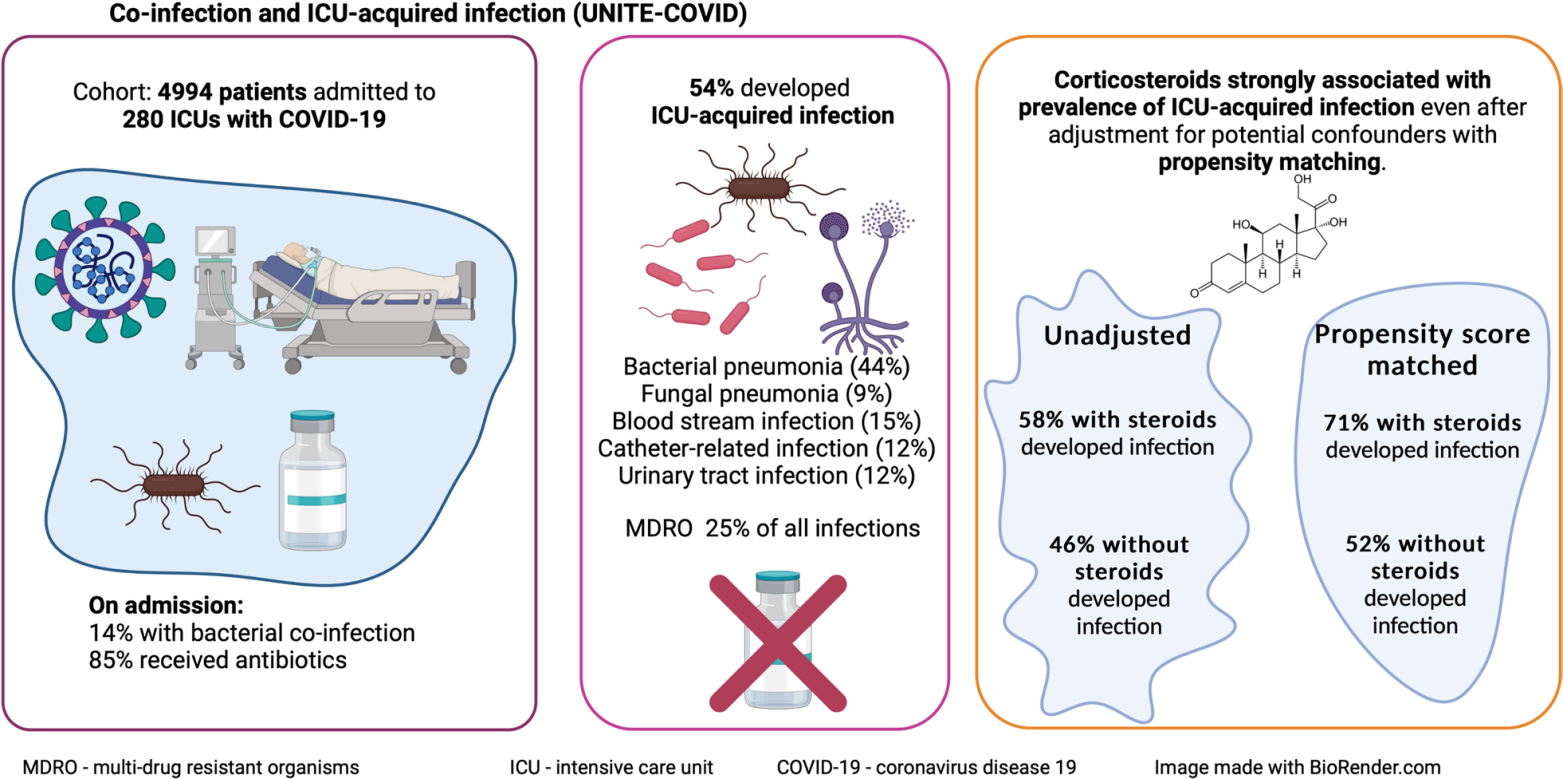

**Supplementary Information:**

The online version contains supplementary material available at 10.1186/s13054-022-04108-8.

## Background

The Coronavirus Disease 2019 (COVID-19) pandemic has led to heighted rates of critical illness and has placed intensive care units (ICU) under unprecedented strain [1]. Although mortality has improved [2, 3], patients continue to experience prolonged admissions to ICU and organ support.

A notable feature critical illness arising from COVID-19 has been the high reported rates of ICU-acquired infections (ICU-AI), most notably ventilator-associated pneumonia (VAP) [4–6] and invasive pulmonary aspergillosis [7, 8]. VAP rates of 40–70% of ventilated patients exceed rates amongst contemporaneous non-COVID-19 patients [4] and historic comparators with influenza [6]. Reported COVID-associated pulmonary aspergillosis (CAPA) rates vary, are likely to be susceptible to the surveillance criteria used [7], but approach those reported for influenza-associated aspergillosis [9] and indeed amongst non-neutropaenic patients with VAP [10]. Other fungal infections such as Mucor mycosis have also been identified [11]. Other ICU-AI, including primary and catheter-associated bacteraemias and urinary tract infections, have been subjected to less attention, but also show elevated prevalence [12, 13]. The use of immunomodulatory therapies in patients with COVID-19 has been widespread, even before definitive evidence was available from randomised trials [14].

Whilst ICU-AI appear to be highly prevalent, exceeding those seen in patients with other viral pneumonias [6], co-infection at presentation appears to be rare [15, 16], reportedly in the range of 3–8%. This differs from influenza where bacterial co-infection is common [17, 18] and mortality is frequently attributed to these co-infecting organisms [19]. Influenced by the experience of influenza and the anticipated risk of co-infection, initial guidelines advocated the use of early broad-spectrum antimicrobials in patients with severe COVID-19 disease [20].

This paper aims to describe the nature of co-infection and ICU-AI in the large UNITE-COVID cohort of patients admitted to critical care across 280 units in 46 countries over 5 continents during the first wave of COVID-19 [1]. In this paper, we investigate factors associated with the development of ICU-AI including the use of corticosteroids.

## Methods

This study is a sub-analysis of the ESICM UNITE-COVID study, which has been extensively described elsewhere [1]. Briefly, UNITE-COVID is a multicenter, international, anonymized observational study carried out in 280 hospitals from 46 countries during ‘wave one’ (defined as 15th of February until 15th of May 2020). Patients who were under intensivist care on the day with the highest number of such patients during wave one were included and followed-up longitudinally for outcomes including ICU-acquired infections, antibiotic use and discharge or death. For each patient, data were collected from hospital admission until discharge.

The study received approval from Ghent University Hospital Ethics committee, registration BC-07826 and appropriate approvals at each participating site in line with local regulations (ClinicalTrials.gov registration: NCT04836065, retrospectively registered April 8th 2021).

### Patients

Inclusion criteria were (1) age ≥ 18 years, (2) cared for by the critical care team in an ICU or other area of the hospital on the above-mentioned date, (3) had PCR (or equivalent technique) confirmed Severe Acute Respiratory Syndrome Coronavirus-2 (SARS-CoV2) infection and (4) had a diagnosis of COVID-19. Patients without a diagnosis of COVID-19 but documented SARS-CoV2 detection were excluded. Patients could only be included once. Informed consent was either obtained or waived according to the local ethics committee’s decision.

### Data

Details of the data collection methods, the collected data, the data curation and the DAQCORD-checklist, are contained in the primary paper [1]. The data curation pipeline and data quality assessment (version 3.1) are publicly available [21]. All data were collected from the day of ICU admission until day 60 following inclusion, with antibiotic use data collected until day 30 after admission. For the purposes of this sub-analysis, we focused on investigator-identified infections at admission and during ICU stay as well as identified multi-resistant microorganisms. ICU-AI were defined as those developing after ICU admission and that were not detected at time of admission to ICU, as identified by the site investigator. Patients with missing data from the relevant field were excluded from that analysis. The case report form is included in the Additional file 1.

### Statistical analysis

Categorical variables are expressed as frequencies (percentages), continuous variables are described with medians with interquartile range (IQR). Differences in categorical variables were calculated using a Pearson chi-squared test. The Mann–Whitney *U* test was used for comparison of non-normally distributed continuous variables. Comparison of non-normally distributed continuous variables with categorical variables was performed by means of the Kruskal–Wallis test. Statistical significance was defined as *p* < 0.05.

Statistical analysis was performed using R Statistical Software (R: A language and environment for statistical computing. R Foundation for Statistical Computing, Vienna, Austria, 4.0.3).

The Strengthening the Reporting of Observational Studies in Epidemiology (STROBE) guidelines for reporting of observational studies in combination with the recommendations to optimize reporting of epidemiological studies on antimicrobial resistance and informing improvement in antimicrobial stewardship (STROBE-AMS) were followed throughout this manuscript. The case report form is viewable as Additional file 1.

We pre-defined factors thought likely to be associated with ICU-AI, namely age, sex, aggregate comorbidity score, use and duration of corticosteroids, need for invasive mechanical ventilation, vasopressors and renal replacement therapy, duration of ventilation and severity of respiratory failure. Severity of respiratory failure was assessed using an ordinal scale of maximal respiratory support similar to that used previously [22] scoring 0 for non-invasive support, 1 for invasive mechanical ventilation, 2 for neuromuscular blockade and 3 for prone ventilation or extra-corporeal membrane oxygenation (ECMO). The combined highest category was used as it was recognised that not all patients with most severe respiratory failure would be referred or accepted for ECMO. We examined univariate association with the development of ICU-acquired infection by pairwise analysis.

To adjust for the pre-defined confounders in our analysis, we used propensity matching [23]. The patient cohort of interest was matched using the R library MatchIt [24] using the “full” matching method [23, 24] as this produced the best match based on standard mean differences. Propensity-matched analysis was restricted to patients who were mechanically ventilated during their ICU stay and who had near-complete data across the fields of interest. Patients with data missing across multiple fields of interest were excluded, where data were missing from one field multiple chained imputation (R library MICE [25]) was employed. We used a 1-to-1 matching of treated to controls in all the analysis except for the tocilizumab treatment analysis where the low number of treated patients allowed a ratio of one treated to three controls without losing match accuracy (Additional file 2: Model Formula S1). Initial analysis excluded those with respiratory co-infection on admission, with inclusion of these patients in a sensitivity analysis.

## Results

Data were available from 4994 patients; demographic and clinical features of these patients have been described previously [1]. Overall 4129 (83%) were mechanically ventilated during their ICU stay, 2325 (47%) within 24 h of admission, whilst a further 1677 (34%) were ventilated later in their stay.

### Co-infection

Bacterial pulmonary co-infection was detected in 716 patients (14%). Despite this, antibacterials were administered to 85% of patients. The immunomodulatory azithromycin made up 19% of all antibacterials, with 35% of all patients being prescribed this drug (Additional file 2: Table S1). Intubation at admission was associated with a significantly greater rate of antibacterial prescription (90% vs 86%, *p* = 0.0014), as was suspicion of co-infection (98% vs 86%, *p* < 0.001). 6% of patients received antifungals at admission (Additional file 2: Table S2). Patients with co-infection at ICU admission had similar pre-ICU hospital length of stay, a median of 1 day (IQR 0–4) with co-infection vs 2 days (IQR 0–4) for those without co-infection (*p* = 0.06).

**Fig. 1:**
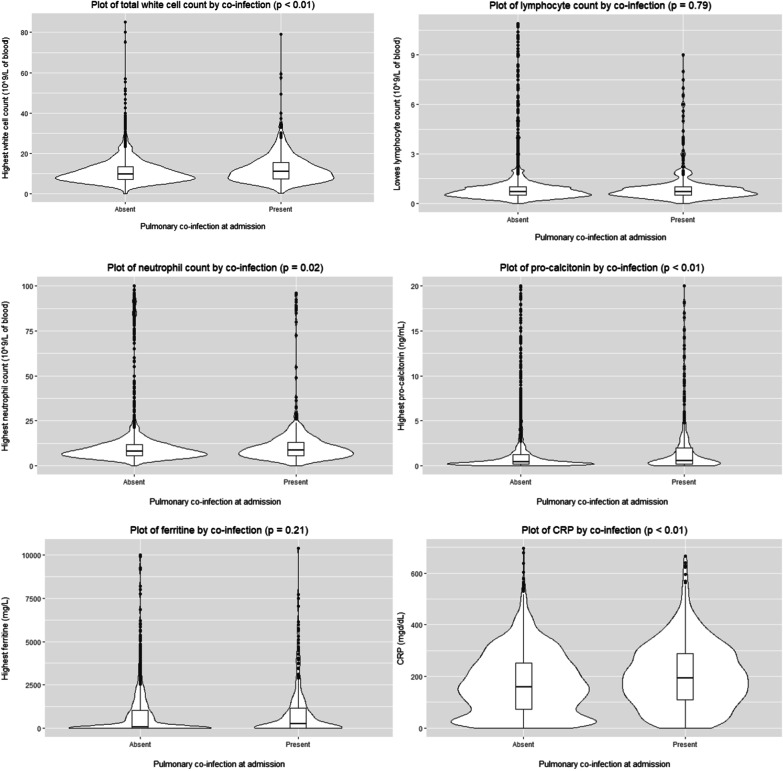
Distribution of values of inflammatory markers within the first 24 h of admission between patients with and without identified co-infection at ICU admission

A comparison of inflammatory parameters between those with and without assessed co-infection at admission is shown in Fig. 1. Although several achieved statistically significant differences, there was substantial overlap between the groups.

### ICU-acquired infections

ICU-AI were common, with 2715 (54%) patients developing a total of 4737 infections (median 2 infections per patient infected). The most common infection was bacterial pneumonia (reported in 77% of patients developing infections), followed by non-catheter-associated bacteraemia (seen in 26% of patients that developed an infection). Fungal respiratory infections were identified in 9% of all patients, and 16% of that developed an infection (Table 1). Of the patients with infection, 25% had an infection with an MDRO. The most prevalent MDRO’s were extended spectrum beta-lactamase producers (ESBL) (7.75%) and Methecillin-resistant *Staphylococcus aureus* (MRSA) (4.75%) (Additional file 2: Table S3).

**Table 1:** Description of ICU-acquired infections identified in patients

Site/class of infection	*N* (% of all infections)	Rate per 1000 ICU days*
Bacterial pulmonary infection	2091 (44%)	22.2/1000 ICU days
Fungal pulmonary infection	447 (9%)	4.7/1000 ICU days
Bacteraemia (not catheter related)	708 (15%)	7.5/1000 ICU days
Catheter/line-associated blood stream infection	563 (12%)	6/1000 ICU days
Urinary tract infection	572 (12%)	6.1/1000 ICU days
Abdominal infection	88 (2%)	0.9/1000 ICU days
Central nervous system infection	20 (0.4%)	0.2/1000 ICU days
Other ICU-acquired infection	248 (5%)	2.6/1000 ICU days

^*^Rate per 1000 ICU days only includes patients with value for length of stay—459 (9%) patients were not included in the rate calculation

Patients received a significant burden of antibiotic exposure during their admission, with a median 7 (IQR 0–17) days alive without antibiotics during the 30-day follow-up. The development of ICU-AI was associated with greater use of antibiotics; a median of 5 (IQR 0–14) antibiotic-free days in those with ICU-AI vs a median of 12 (IQR 0–23) in those without infection.

### Factors associated with ICU-AI

Factors evaluated for association with ICU-AI are shown in Table 2. ICU-AI was associated with longer ICU length of stay (median 24 days (IQR 16–35) days vs 11 days (IQR 6–17) (*p* < 0.001), as was infection with MDRO (29 days (IQR 19–43) days for MDRO vs 23 days (IQR 15–33) for those with non-MDRO infection (*p* < 0.001).

**Table 2:** Demographic and clinical factors assessed for association with development of ICU-acquired infections, median (IQR) values shown for continuous and ordinal variables

Parameter	ICU-acquired infection	No ICU-acquired infection	*p* value
Age	62 (54–70)	62 (52–71)	0.975
Percentage male	73%	69%	0.003
Comorbidity score	1 (0–2)	1 (0–1)	0.0004
Percentage receiving steroid treatment	58%	43%	< 0.001
Ventilation severity score	3 (2–3)	2 (1–3)	< 0.001
Mechanically ventilated at any time	97%	72%	< 0.001
Ventilation duration (days)	21 (14–31)	10 (6–15)	< 0.001
Vasopressor/inotrope at any time	50%	25%	< 0.01
Renal replacement therapy at any time	18%	6%	< 0.01

**Fig. 2:**
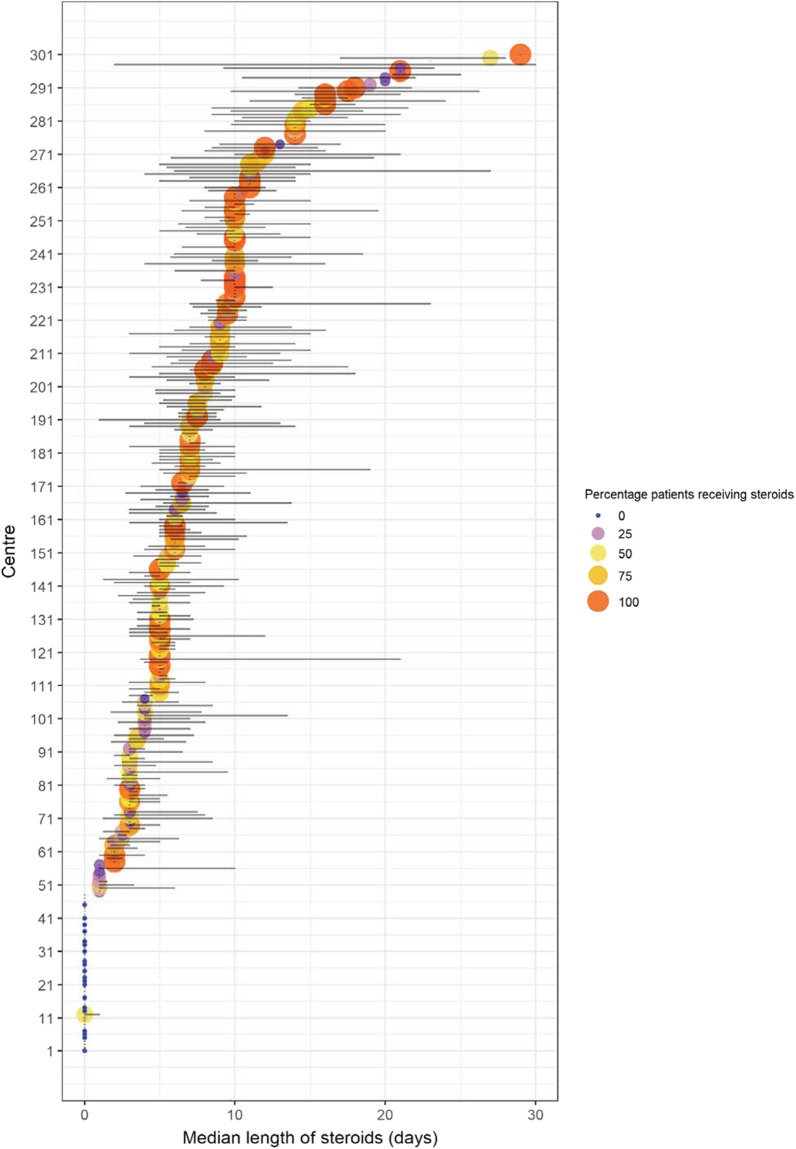
Forest plot of steroid treatment by centre: Colour and dot size represent the percentage of overall patients that received steroid treatment, the forest plot shows the median and IQR of length of steroid treatment. Centres are ordered by the median length of time patients receive steroids

Receipt of antibiotics at admission was associated with development of subsequent infection (58% vs 46% of those not receiving antibiotics, *p* < 0.001), but was not significantly associated with development of MDRO infection (24% vs 20%, *p* = 0.2). Fewer antimicrobial-free days were associated with a higher prevalence of MDRO infection (median antimicrobial-free days 2 (IQR 0–9) for patients with an MDRO infection vs 6 days (IQR 0–15) for patients without an MDRO infection, *p* < 0.01). Corticosteroid use was higher amongst patients developing infection in ICU (58% vs 43%). Steroid use was heterogenous across centres (Fig. 2) with the dominant indications being hyperinflammation and pneumonitis, which together made up 65% of indications recorded (Additional file 2: Table S4). Shock was the indication in 16% of cases.

### Propensity-matched analysis.

**Fig. 3:**
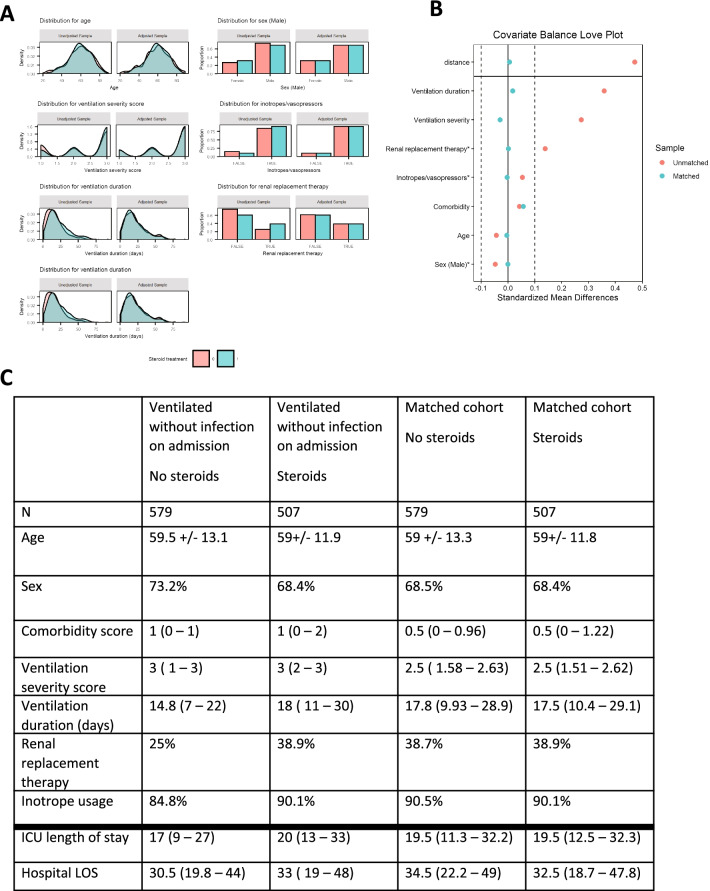
Propensity score (PS) matching for corticosteroid matching parameters compared to the input cohort. **A** density plots and histograms showing the effect of PS matching on distributions. **B** Covariance balance ‘love’ plot illustrating the effect of PS on standardised mean difference. **C** Numeric summary statistics following PS matching. Reporting percentages for categorical variables, mean for non-skewed parameters, and median for skewed parameters. Last two entries below the thick line are outcome measures and were not used for matching

Following the finding of an increased risk of infection amongst patients receiving corticosteroids in univariate analysis, we undertook a propensity-matched analysis to further understand this relationship, undertaking matching for pre-defined factors thought to be associated with infection (Table 2). Heterogeneity of steroid use between units extended to both the percentage of patients receiving steroids in each centre and the duration of therapy (Fig. 2). Additional file 2: Fig. S1 shows the selection of the matched cohort. Using 1:1 matching, 1086 were entered into this analysis, and matching was highly effective (Fig. 3), with absolute standardised mean differences after matching all less than 0.1. The calculated pseudo-R^2^ was 0.33 (details in Additional file 2).

In the matched cohort, the relationship between steroids and infection was maintained: 71% of those receiving steroids developed ICU-AI vs 52% of those not in receipt of steroids (*p* < 0.001). The main indications for steroids in the propensity-matched cohort were hyper-inflammation and shock (Additional file 2: Table S5). Steroid use was also associated with MDRO infections (17% of infections in patients on steroids were with MDRO organisms, vs 5% of those not on steroids (*p* < 0.001). Duration of steroid administration was also associated with ICU-AI in this analysis, with those developing ICU-AI receiving steroids for a median of 8 days (IQR 3–14)) vs 5 days (IQR 3–10) for those not developing infection. The median time spent in ICU before steroid administration was 2 days (IQR 0–8) in patients who did not develop an infection in ICU and 6 days (IQR 1–13) in those who did. The mortality rate was higher in the steroid treatment cohort with 37% compared to 30% in the non-steroid cohort (*p* = 0.02).

As a sensitivity analysis, inclusion of those who had co-infection on admission did not significantly alter the results of the propensity-matched analysis (Additional file 2: results).

Rates of tocilizumab use were much lower than corticosteroids, with only 132 patients receiving this. In a 1:1 match using the same matching variables and matching method, tocilizumab use was not associated with an increase in the risk of ICU-AI: 56% of patients without tocilizumab developed infection vs 46% of those receiving this agent (*p* = 0.04). Details of the matching are shown in Additional file 2: Figs. S2 and S3.

## Discussion

This study describes the largest ICU cohort with COVID-19 so far reported and covers patients from a wide range of countries. The rate of co-infection, at 14%, is above the 3–10% reported in other studies [15, 16, 18, 26] including those that were restricted to critically ill patients [26]. Although there was a marginal effect of co-infection on the use of antibiotics at admission, the great majority of patients received antibiotics despite the absence of assessed co-infection. At the time of data collection, guidelines indicated that broad spectrum antibiotics should be administered to all patients with severe disease [20].

The finding of high rates of ICU-AI, with over half of all patients developing such infections, is consistent with previous reports from critically ill COVID-19 patients which have largely focussed on ventilator-associated pneumonia [4–6], and bacteraemia [12, 13], notably also the predominant infections encountered in this cohort. Whilst debate continues regarding the true incidence of CAPA [27, 28], in this study we found fungal pulmonary infections complicated the stay of 9% of all patients. This is a lower prevalence than some reports [8], but may reflect underdiagnosis as at the time of data collection CAPA was a novel diagnosis [7]. The rate of ICU-AI reported far-exceeds pre-pandemic rates reported by the European Centres for Disease Control (ECDC) [29]. In 2017, ECDC reported rates of ICU-acquired infection were 8.3% for all patients staying more than 2 days in ICU, with pneumonia complicating 6% of stays, bloodstream infections (BSI) 4% and urinary tract infections (UTI) 2% [29]. Whilst diagnostic uncertainty caused by tracheal colonisation and viral pneumonitis may confound the incidence of ICU-acquired pneumonia, such uncertainty is unlikely to be present with infections at other sites. Studies from multiple units which have used rigorous diagnostic criteria have found a similarly high rate of bacterial pneumonia [4–6] reporting prevalences of 44 to 50%. We cannot be certain that some identified ICU-AIs were not, in-fact, missed co-infections, and this could influence the relationship between initial antibiotics and subsequent detected ICU-AI. However, previous studies of ICU-AI have shown clinicians can readily distinguish the two [30, 31]. This is unlikely to alter the results of our findings, as including patients with identified co-infection did not substantially alter the results from the propensity-score-matched analysis (Additional file 2: results). The high rate of secondary infection seen in this current cohort was reflected in associated exposure to antibiotics, and strikingly, a substantial proportion of antibiotic use was not targeted towards either co-infection or ICU-AI. MDRO were encountered in a quarter of all those developing ICU-AI, similar to the prevalence reported in other large surveys of infection in ICU [32]. The association between duration of antimicrobial therapy and development of MDROs may reflect the selective pressure of prolonged antimicrobial infection but may also reflect the perceived need for prolonged antimicrobial therapy in patients who develop MDRO infections.

The reasons for the high rates of ICU-AI amongst patients with COVD remain uncertain and cannot be completely explained by prolonged duration of organ support [4, 6]. The ECDC 2017 report notes median incident density of 3.7/1000 ICU days (IQR 0.8–4.9) for pneumonia, 1.9/1000 ICU days (IQR 0.4–3.1) for all BSIs and 2.4/1000 ICU days (IQR 0–3.7) for UTI [29]. The incident density shown in Table 1 is in considerable excess of these. In this study, we identified a significant association between the use of corticosteroids and the development of such infections and infections with MDRO, which was maintained after adjustment for potential confounders. As an observational study, however, we cannot exclude residual confounding as a cause of this association. In this study, corticosteroids were not associated with increased survival, so it is unlikely that their use increased infection due to patients surviving long enough to develop an infection in ICU. The finding of increased unadjusted mortality amongst patients receiving steroids may reflect differences between patients selected for corticosteroid therapy, although other reports have noted similar effects even after adjusting for clinical variables [33, 34].

The literature concerning the effect of corticosteroids on ICU-AIs is divergent, with reports of both increased [35] and decreased infection rates [36]. In the randomised trials examining the use of corticosteroids in COVID-19 [37–41] an association was not found with secondary infections. However, the largest of these studies [38, 41] did not specifically look for secondary infections. Two smaller studies [39, 40] reported no significant differences, although the overall rates of secondary infection were lower than those reported here and elsewhere in the literature [4–6, 12, 13]. Our study is observational, and so we cannot exclude unmeasured cofounders. However, it reports data from ‘wave one’ before the announcement of the results of the landmark RECOVERY trial [38] and corticosteroid use varied markedly between centres (Fig. 2). Furthermore, a wide range of steroid doses and durations of therapy were deployed early in the pandemic. It may be that prolonged and high-dose courses of corticosteroids explain why our results diverge from those reported in clinical trials [39, 40]. Other observational studies have found a relationship between corticosteroid use and subsequent pneumonia in single centre [42] and multi-centre cohorts [34, 43], with the Dupuis and colleagues study noting a relationship between steroid dose and infection risk [34]. It is possible that steroids were prescribed because of secondary infection *i.e.* a reverse causal relationship, though we did not find an excess of shock amongst patients receiving steroids who developed secondary infection (Additional file 2: Table S5). It was, perhaps, surprising to find that tocilizumab was not associated with an increased risk of ICU-acquired infection and indeed the effect seen was in the opposite direction. We note a similar finding recently reported in a single centre study of catheter-related blood stream infections, where dexamethasone but not tocilizumab was associated with increased risk of infection [44]. It is possible that this finding resulted from failure to diagnose infection due to supressed inflammatory responses, most especially C-reactive protein and procalcitonin concentrations [45]. Alternatively, if tocilizumab was reserved for later in patient stays, it may have been withheld in patients in whom there was suspicion of secondary infection; however, lack of data on the relative timing of these events makes these hypotheses speculative.

This study has a number of strengths, among them that it is the largest study of critically ill patients with COVID-19 reported to date and provides data from a wide range of geographic locations and healthcare settings. However, the data collected were limited by the feasibility of collecting data from hundreds of geographically diverse sites during the early phase of the pandemic. As such we had to rely on investigator ascertainment of the presence of infection, which may not have been standard across sites and did not collect details of microbiological cultures beyond the presence or absence of specific MDROs. That the rates of ICU-AI were similar to those reported from smaller, but more rigorously controlled studies are reassuring [4–6, 11, 12], and patterns seen were similar across all infections, as well as the more objective measures of MDRO rates and antibiotic use. With widespread use of antibiotics noted, it is possible that these would mask the detection of ICU-AI and that the rates of ICU-AI are actually higher than we report, although this is unlikely to alter the relationships seen with the therapeutic agents examined. We did not collect data on formulation or dose of corticosteroid used and therefore cannot assess the impact that variations in these may have had.

## Conclusions

In conclusion, this study has demonstrated widespread use of antibiotics in critically ill patients during the first wave of COVID-19. Although this use may appear indiscriminate, their use at admission was in line with guidelines extant at the time of patient inclusion [20]. Admissions with bacterial co-infection were relatively rare, and there was significant potential to limit antibiotic use at this point. Although ICU-AI were common, driving antibiotic use, this use extended beyond patients with secondary infections, indicating potential for further reduction in antibiotic utilisation. The finding of an association between corticosteroid use and ICU-AI requires further exploration, as do strategies for infection diagnosis in the presence of concurrent anti-cytokine therapies.

## Supplementary Information

**Additional file 1:** Case report form.

**Additional file 2:** Supplemental data.

## Data Availability

The datasets used and/or analysed during the current study are available from the corresponding author on reasonable request. The data curation pipeline and data quality assessment (version 3.1) are publicly available https://doi.org/10.5281/zenodo.6063905

## Abbreviations

BSI: Blood stream infection
COVID-19: Coronavirus disease 19
CAPA: COVID-associated pulmonary aspergillosis
ECMO: Extra-corporeal membrane oxygenation
ECDC: European Centres for Disease Control
ESBL: Extended spectrum beta-lactamase producers
ICU: Intensive care unit
ICU-AI: Intensive care unit-acquired infection
MDRO: Multi-drug resistant organisms
MRSA: Methecillin-resistant *Staphylococcus aureus*
SARS-CoV2: Severe Acute Respiratory Syndrome Coronavirus-2
STROBE: The Strengthening the Reporting of Observational Studies in Epidemiology
STROBE-AMS: The Strengthening the Reporting of Observational Studies in Epidemiology antimicrobial resistance and informing improvement in antimicrobial stewardship
UTI: Urinary tract infection
VAP: Ventilator-associated pneumonia
