# Supplementary material for: Co-infection and ICU-acquired infection in COVID-19 ICU patients: a secondary analysis of the UNITE-COVID data set
Source: Crit Care. 2022 Aug 3;26:236. doi: 10.1186/s13054-022-04108-8 (PMC9347163; doi:10.1186/s13054-022-04108-8)
Supplement: Supplementary file 1 — Additional file 1: Case report form. [file 13054_2022_4108_MOESM1_ESM.pdf]

EUROPEAN SOCIETY OF INTENSIVE CARE MEDICINE COVID-19 Project (UNITE-COVID)

PATIENT CRF
Version 28JUN2021

CLINICAL INCLUSION CRITERIA (all required)

Proven infection with SARS-CoV2 ☐ Yes ☐ No **Critically ill with COVID-19** ☐ Yes ☐ No

DEMOGRAPHICS

Sex ☐ Male ☐ Female ☐ Not specified **Age** [___][___][___] years **Height** [___][___][___] cm **Weight** [___][___][___] kg
Healthcare Worker? ☐ Yes ☐ No ☐ Unknown **Pregnant?** ☐ Yes ☐ No ☐ Unknown ☐ N/A

Episode information

LOS in hospital prior to ICU admission [ ][ ] days **Patient admitted in surge capacity bed** ☐ Yes ☐ No
Interval start of symptoms – hospital admission [ ][ ] days ☐ Unknown

COMORBIDITIES *(existing prior to admission)*

Chronic cardiac disease *(not hypertension)*	☐ Yes ☐ No ☐ Unknown	Chronic liver disease	☐ Yes ☐ No ☐ Unknown
Arterial hypertension	☐ Yes ☐ No ☐ Unknown	Chronic neurological disorder	☐ Yes ☐ No ☐ Unknown
Chronic pulmonary disease	☐ Yes ☐ No ☐ Unknown	Diabetes	☐ Type I ☐ Type II ☐ No ☐ Unknown
Asthma	☐ Yes ☐ No ☐ Unknown	Malignant neoplasm	☐ Yes ☐ No ☐ Unknown
Chronic kidney disease	☐ Yes ☐ No ☐ Unknown	Immunosuppression	☐ Yes ☐ No ☐ Unknown

HIV ☐ Yes-on ART ☐ Yes-not on ART ☐ No ☐ Unknown

PRE-ADMISSION & CHRONIC MEDICATION Did the patient receive any of these regularly in 14 days prior to admission?

ACE inhibitors	☐ Yes ☐ No ☐ Unknown	Anticoagulation	☐ Yes ☐ No ☐ Unknown
Angiotensin II receptor blockers	☐ Yes ☐ No ☐ Unknown	Antiplatelet therapy	☐ Yes ☐ No ☐ Unknown

ICU Admission data (in case a patient was referred from another ICU, these data should be from the current admission)

ICU Admission diagnosis ☐ Respiratory failure due to COVID-19 ☐ Other complication of COVID-19 ☐ Other diagnosis
If other than Respiratory failure, please add detail: ________________________ ☐ **Referral from another ICU**
Was a thromboembolic complication present on admission? ☐ DVT ☐ PE ☐ Other ☐ None documented
Did the patient receive respiratory support before ICU admission? ☐ Yes ☐ No ☐ Unknown
If YES, which type of support? ☐ Standard oxygen ☐ HFNO ☐ CPAP ☐NIV
Total duration of support (HFNO, CPAP and/or NIV) before admission[___][___] days
Clinical and lab parameters on admission (record highest/lowest value in 24 hours following ICU admission)
Highest Temperature (ºC) [___][___],[___] Highest total white cell count (10^9/L of blood) [___][___][___]
Highest neutrophil count (10^9/L of blood) [___][___][___] Lowest Lymphocyte count (10^9/L of blood[___][___][___]
Highest C-reactive protein (mg/L) [___][___][___] Highest pro-calcitonin (ng/mL) [___][___],[___]
Highest ferritin (mg/L) [___][___][___][___][___] Highest hs-troponinT (ng/mL) [___][___],[___]
Clotting parameters on admission (record highest/lowest value in 24 hours following ICU admission)
Highest fibrinogen (g/L) [___][___],[___] Highest aPTT (sec) [___][___][___] Highest D-dimers (ng/mL) [___][___][___][___][___]
Lowest platelet count (10^9/L of blood) [___][___][___] Highest prothrombin time (sec) [___][___][___]

COMPLICATIONS DURING ICU STAY: At any time during ICU stay did the patient experience:

Cardiac arrhythmia req therapy	☐ Yes ☐ No ☐ Unknown	Prolonged delirium	☐ Yes ☐ No ☐ Unknown
Sepsis induced myocardiopathy	☐ Yes ☐ No ☐ Unknown	Seizure	☐ Yes ☐ No ☐ Unknown
Stress myocardiopathy	☐ Yes ☐ No ☐ Unknown	Pressure sores – facial (prone)	☐ Yes ☐ No ☐ Unknown
Myocarditis	☐ Yes ☐ No ☐ Unknown	Pressure sores – other	☐ Yes ☐ No ☐ Unknown
Pericardial effusion	☐ Yes ☐ No ☐ Unknown	Acute kidney injury	☐ Yes ☐ No ☐ Unknown
Pneumothorax	☐ Yes ☐ No ☐ Unknown	Tube obstruction	☐ Yes ☐ No ☐ Unknown
Atelectasis	☐ Yes ☐ No ☐ Unknown	Accidental extubation	☐ Yes ☐ No ☐ Unknown

MEDICATION DURING ICU STAY: While hospitalized in the ICU were any of the following administered?

Antivirals? ☐Yes ☐No ☐Unknown **If yes:** ☐Ribavirin ☐Lopinavir/Ritonavir ☐Neuraminidase inhibitor ☐ Remdesivir
Other: ☐Interferon alpha ☐Interferon beta ☐ Tocilizumab ☐ Anakinra ☐ Convalescent plasma
Corticosteroid? ☐Yes ☐No ☐Unknown **If yes, total duration:** [___][___]days and **interval after admission**[___][___] days
If yes, indication: ☐ Shock ☐ Hyperinflammation ☐ Pneumonitis ☐ Pre-existing condition ☐ Other
Antimalarial agent? ☐Chloroquine ☐Hydroxychloroquin ☐None **If yes, total duration:** [___][___] days
Was the patient included in a clinical trial (drug) ☐Yes ☐No ☐Unknown

SUPPORTIVE CARE DURING ICU STAY: During hospitalization, did the patient receive/undergo:

Sedation? ☐ Yes ☐ No ☐ Unknown **If yes, total duration:** [___][___] days
Renal replacement therapy (RRT) or dialysis? ☐Yes ☐No☐ Unknown **If yes, total duration:** [___][___] days
If yes, method: ☐ CRRT ☐ Intermittent ☐ Peritoneal dialysis ☐ Mixture **RRT method outside unit's usual practice?** ☐Yes ☐No
Other form of extracorporeal blood purification ? ☐ Yes ☐ No **If yes:** ☐ hemoperfusion ☐ hemoadsorption ☐ other
Inotropes/vasopressors? ☐Yes ☐No ☐ Unknown **If yes, total duration:** [___][___] days
Was the patient tracheostomized? ☐Yes ☐No ☐Before this admission **If yes, at which day of mechanical ventilation** [___][___]

If yes: ☐Surgical ☐Dilatative/percutaneous ☐Unknown

Clinical and lab parameters during ICU stay (record highest/lowest value DURING ICU admission)

Highest Temperature (ºC) [___][___],[___]
Highest total white cell count (10^9/ml of blood) [___][___][___]
Highest neutrophil count (10^9/ml of blood) [___][___][___]
Lowest Lymphocyte count (10^9/ml of blood[___][___][___]
Highest ferritin (mg/L) [___][___][___]
Highest hs-troponinT (ng/mL) [___][___],[___]

OUTCOME – to be evaluated at 60 days after admission to the ICU (based on information in ICU and hospital records)

Outcome: ☐Still in ICU ☐Hospitalized ☐Transfer to other facility ☐Discharged alive ☐Death ☐Palliative discharge ☐Unknown

If no longer in ICU, ICU admission duration: [___][___] days **If dead, did patient die in the ICU?** ☐Yes ☐No

If discharged alive/transfer, hospital admission duration: [___][___] days **If discharged alive, was patient still on RRT?** ☐Yes ☐No

DOMAIN Respiratory

Was the patient intubated at ICU admission? ☐Yes ☐No **If not, was the patient intubated during the ICU stay?** ☐Yes ☐No

If the patient was intubated during the ICU stay, how many days after admission [___][___] days

During ICU stay, did the patient receive any of the following:

Non-invasive ventilation? *(e.g. BIPAP, CPAP) ☐Yes ☐No ☐Unknown* ***If yes, duration (if intubated, before intubation):*** *[___][___] days*

HFNC? *☐Yes ☐No ☐Unknown* ***If yes, duration (if intubated, before intubation):*** *[___][___] days*

Invasive ventilation *(Any) ☐Yes ☐No ☐Unknown* ***If yes, total duration:*** *[___][___] days*

Extracorporeal support (ECMO)? *☐Yes ☐No ☐Unknown* ***If yes, total duration:*** *[___][___] days*

Prone position? *☐Yes ☐No ☐Unknown* ***If yes, duration intubated:*** *[___][___] days* ***If yes, duration not intubated:*** *[___][___] days*

Neuromuscular blockers *☐Yes ☐No ☐Unknown* ***If yes, total duration:*** *[___][___] days*

Which type of support did the patient receive after extubation/weaning? ☐ Standard oxygen ☐HFNO ☐CPAP ☐NIV

For intubated patients: please indicate the ventilatory settings on the first day after intubation:

Mode: *☐VCV ☐PCV ☐BIPAP ☐APRV ☐PSV* ***Tidal Volume (mL)*** *[___][___][___]* ***PEEP (cmH_2O):*** *[___][___]*

FiO_2 (%): *[___][___][___]* ***P/F ratio:*** *[___][___][___]* ***$PaCO_2$ (mmHg):*** *[___][___][___]* ***Driving Pressure (cmH_2O):*** *[___][___]*

Ventilator not routinely used in your ICU? ☐Yes ☐No **How was the weaning process?:** ☐Normal ☐Difficult ☐Prolonged

Was the patient reintubated after initial extubation? ☐Yes ☐No ☐Unknown

DOMAIN Coagulation

Clotting parameters during ICU stay (record highest/lowest value DURING ICU admission)

Lowest fibrinogen (g/L) [___][___],[___]
Highest D-dimers (ng/mL) [___][___][___][___][___]
Lowest platelet count (10^9/ml of blood) [___][___][___]
Highest platelet count (10^9/ml of blood) [___][___][___]
Highest prothrombin (time (sec) [___][___][___] Highest aPTT (sec) [___][___][___] Highest ferritin(mg/L) [___][___][___]][___][___]

DVT prophylaxis (during first 24h of admission: drug and daily dose)

Antiplatelet prophylaxis (during first 24h of drug, daily dose)

Life-threatening hemorrhagic complications (e.g. shock, airway compromise, intracranial mass effect, etc.): ☐ Yes ☐No

Source of bleeding ☐Lines ☐GI ☐Respiratory tract ☐CNS ☐Other **Number of Packed cells transfused**: [___][___]

Thromboembolic complications ☐DVT ☐PE ☐Myocardial infarction ☐Limb ischemia ☐ Stroke

Therapeutic anticoagulation ☐ Yes ☐ No ; if yes ☐ UFH ☐ LMWH ☐ other ; interval after ICU admission [___][___]

Indication for anticoagulation ☐DVT ☐PE ☐Myocardial infarction ☐limb ischemia ☐Line or filter clot ☐Prophylaxis ☐Previous condition

DOMAIN Infection

Did the patient receive the following within 24 hours of ICU admission?

Antibiotics ☐ Yes ☐ No ☐ Unknown if yes insert codes here [___][___] - [___][___] - [___][___] - [___][___]
Antifungal ☐ Yes ☐ No ☐ Unknown if yes insert codes here [___][___] - [___][___] - [___][___] - [___][___]
Was bacterial pulmonary co-infection present at admission? ☐ Yes ☐ No
Did the patient develop an infection at any point during ICU stay ☐ Yes ☐ No **If yes, severity** ☐ Sepsis ☐ Septic shock

Bacterial pulmonary infection	☐ Yes ☐ No	Urinary tract infection	☐ Yes ☐ No
Fungal respiratory infection	☐ Yes ☐ No	CNS infection	☐ Yes ☐ No
Abdominal infection	☐ Yes ☐ No	Other infection	☐ Yes ☐ No
Bacteremia (not catheter related)	☐ Yes ☐ No	CLABSI	☐ Yes ☐ No

Was an MDR pathogen involved? ☐ Yes ☐ No **If yes, specify:** ☐ MRSA ☐ VRE ☐ MDR-PA ☐ CRE ☐ ESBL ☐ Acinetobacter
Days alive without anti-microbial therapy at day 30 [___][___]

DOMAIN Rehabilitation

Was the patient mobilized in the first 72h of ICU stay? ☐ Yes ☐ No ☐ Unknown If yes, highest achieved IMS: [___][___]

Was the patient mobilized in the first 72h of mechanical ventilation? ☐ Yes ☐ No ☐ Unknown If yes, highest IMS: [___][___]

Was the patient mobilized during total ICU stay? ☐ Yes ☐ No ☐ Unknown If yes, highest achieved IMS: [___][___]

If the patient was on ECMO, highest achieved IMS while on ECMO: [___][___]

Information for completing the UNITE COVID19 patient CRF
