# Supplementary material for: Co-infection and ICU-acquired infection in COVID-19 ICU patients: a secondary analysis of the UNITE-COVID data set
Source: Crit Care. 2022 Aug 3;26:236. doi: 10.1186/s13054-022-04108-8 (PMC9347163; doi:10.1186/s13054-022-04108-8)
Supplement: Supplementary file 2 — Additional file 2: Supplemental data. [file 13054_2022_4108_MOESM2_ESM.docx]

**Supplemental data**

**Co-infection and secondary infection in COIVD-ICU: a secondary analysis of the UNITE-COVID data set.**

Andrew Conway Morris, Katharina Kohler, Thomas De Corte^4^, Ari Ercole, Harm-Jan De Grooth, Paul Elbers, Pedro Povoa, Rui Morais, Despoina Koulenti, Sameer Jog, Nathan Nielsen, Alasdair Jubb, Maurizio Cecconi, Jan De Waele for the ESICM UNITE COVID investigators.

Table S1: Antibacterials prescribed within first 24 hours of ICU admission

| **Prescribed antimicrobial** | **Number of prescriptions** | **Percentage of total antimicrobial prescriptions (%)** | **Percentage of patients on antimicrobials receiving this antimicrobial (%)** |
| --- | --- | --- | --- |
| Azithromycin | 1450 | 19% | 35% |
| Ceftriaxone | 1443 | 19% | 35% |
| Piperacillin-tazobactam | 858 | 11% | 21% |
| Meropenem | 526 | 7% | 13% |
| Coamoxiclav | 517 | 7% | 12% |
| Clarithromycin | 408 | 5% | 10% |
| Other cephalosporin | 352 | 5% | 8% |
| Levofloxacin | 322 | 4% | 8% |
| Vancomycin | 306 | 4% | 7% |
| Other antimicrobial | 216 | 3% | 5% |
| Linezolid | 167 | 2% | 4% |
| Other penicillin | 154 | 2% | 4% |
| Ciprofloxacin | 148 | 2% | 4% |
| Cefuroxime | 112 | 2% | 3% |
| Cefepime/cefpirome | 110 | 1% | 3% |
| Teicoplanin | 73 | 1% | 2% |
| Amikacin | 60 | 1% | 1% |
| Ceftazidim | 60 | 1% | 1% |
| Ampicillin | 52 | 1% | 1% |
| Imipenem | 51 | 1% | 1% |
| Other quinolone | 38 | 1% | 1% |
| Cefazolin | 34 | 0.4% | 0.8% |
| Gentamicin | 34 | 0.4% | 0.8% |
| Tigecycline | 34 | 0.4% | 0.8% |
| Cotrimoxazole | 27 | 0.4% | 0.7% |
| Erythromycin | 27 | 0.4% | 0.7% |
| Oxa/cloxa/flucloxacillin | 25 | 0.3% | 0.6% |
| Metronidazole | 22 | 0.3% | 0.5% |
| Other glycopeptide | 17 | 0.2% | 0.4% |
| Benzylpenicillin | 16 | 0.2% | 0.4% |
| Ertapenem | 15 | 0.2% | 0.4% |
| Aztreonam | 13 | 0.2% | 0.3% |
| Other aminoglycosides | 8 | 0.1% | 0.2% |
| Other betalactams | 4 | 0.05% | 0.1% |
| Daptomycin | 3 | 0.04% | 0.07% |
| Other carbapenem | 3 | 0.04% | 0.07% |
| Tobramycin | 3 | 0.04% | 0.07% |
| Doripenem | 2 | 0.03% | 0.05% |
| Type of antimicrobial not provided |  |  | 1.05% |

Table S2: antifungals prescribed within first 24 hours of ICU admission

| **Prescribed antifungal** | **Number of prescriptions** | **Percentage of total antifungal prescriptions (%)** | **Percentage of patients on antifungals receiving this antifungal (%)** |
| --- | --- | --- | --- |
| Fluconazole | 67 | 24.01% | 24.01% |
| Echonocandin | 41 | 14.7% | 14.7% |
| Voriconazole | 39 | 13.98% | 13.98% |
| Other antifungal | 30 | 10.75% | 10.75% |
| Amphotericin B | 16 | 5.73% | 5.73% |
| Amphotericin lipid formulation | 3 | 1.08% | 1.08% |
| Antifungal administration recorded but specific drug not indicated |  |  | 36.92% |

Table S3:Multi-drug resistant organisms cultured from patients with secondary infection

| **Organism/Resistance mechanism** | N (%) |
| --- | --- |
| Extended spectrum beta-lactamase secretor | 205 (31%) |
| Methicillin resistant *Staphylococcus aureus* | 123 (19%) |
| Multi-drug resistant *Acinetobacter* | 116 (18%) |
| Multi-drug resistant *Pseudomonas aeruginosa* | 115 (18%) |
| Vancomycin resistant *Enterococci* | 57 (9%) |
| Carbapenem resistant Enterobacterales | 36 (6%) |

Table S4: indications for corticosteroid use in the whole cohort

| Indication | Number of patients (% of those receiving steroids) | Percentage of total population (4994 participants) |
| --- | --- | --- |
| Hyper-inflammation | 1034 (42%) | 20.7% |
| Pneumonitis | 573 (23%) | 11.5% |
| Shock | 385 (16%) | 7.7% |
| Other | 326 (13%) | 6.5% |
| Pre-existing condition | 131 (5%) | 2.6% |
| Unknown | 15 (1%) | 0.3% |
| No steroids received | 2530 (NA) | 50.7% |

Table S5: Indications for corticosteroid use in the propensity matched cohort

| Indication for steroids | All patients with steroid treatment | Patients with steroids treatment and no infection | Patients with steroid treatment and with secondary infection |
| --- | --- | --- | --- |
| Hyper-inflammation | 30.6% | 30.6% | 30.5% |
| Shock | 24.0% | 22.4% | 24.7% |
| Other | 19.3% | 24.5% | 17.2% |
| Pneumonitis | 17.8% | 17.0% | 18.1% |
| Pre-existing condition | 7.7% | 4.0% | 9.1% |
| unknown | 0.6% | 1.4% | 0.3% |
|  |  |  |  |
| Median time to steroids (in ICU) | 5 ( 0 – 10.5) | 2 (0 – 8) | 6(1 – 13) |
|  |  |  |  |
| Median duration of steroids | 7 (3 – 12) | 5 (3 – 10) | 8 (3 – 14) |

**Sensitivity analysis for propensity score matched effect of steroids on ICU-acquired infection**

Inclusion of those who had co-infection on admission did not significantly alter the results of the propensity matched analysis. 74% of patients receiving steroids and 57% of those not developed infections in ICU, *p*<0.001, with a similar pattern seen in MDRO with 16% of steroid recipients developing MDRO infections vs 7% of non-recipients, *p*<0.001).

**Model:**

Formula S1.

Model corticosteroid administration based on:

comorbidity + ventilation severity + sex + age + ventilation duration + renal replacement therapy + inotrope usage using the “full method” and a ratio of 1:1

Pseuso-R^2^ was calculated to be 0.33 using the “rms” package in R.

**Supplemental figures**

Figure S1: Cohort selection chart for propensity score matching to investigate the effect of steroids on secondary infection.


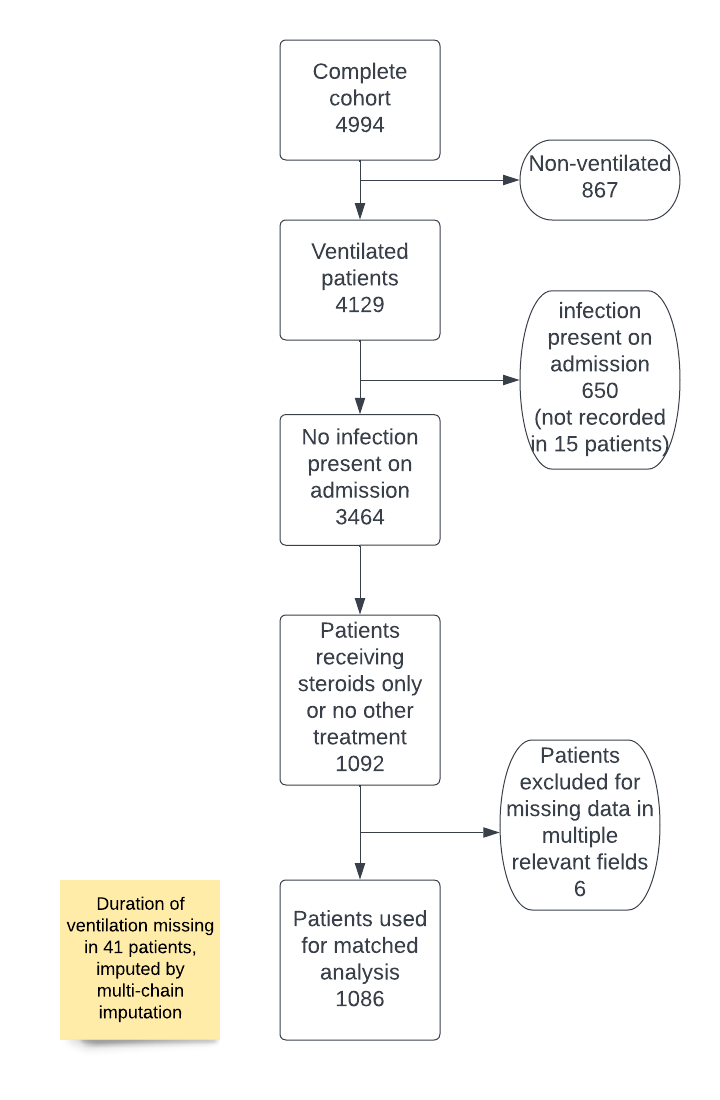


Figure S2 : Cohort selection chart for propensity score matching to investigate the effect of tocilizumab on secondary infection.


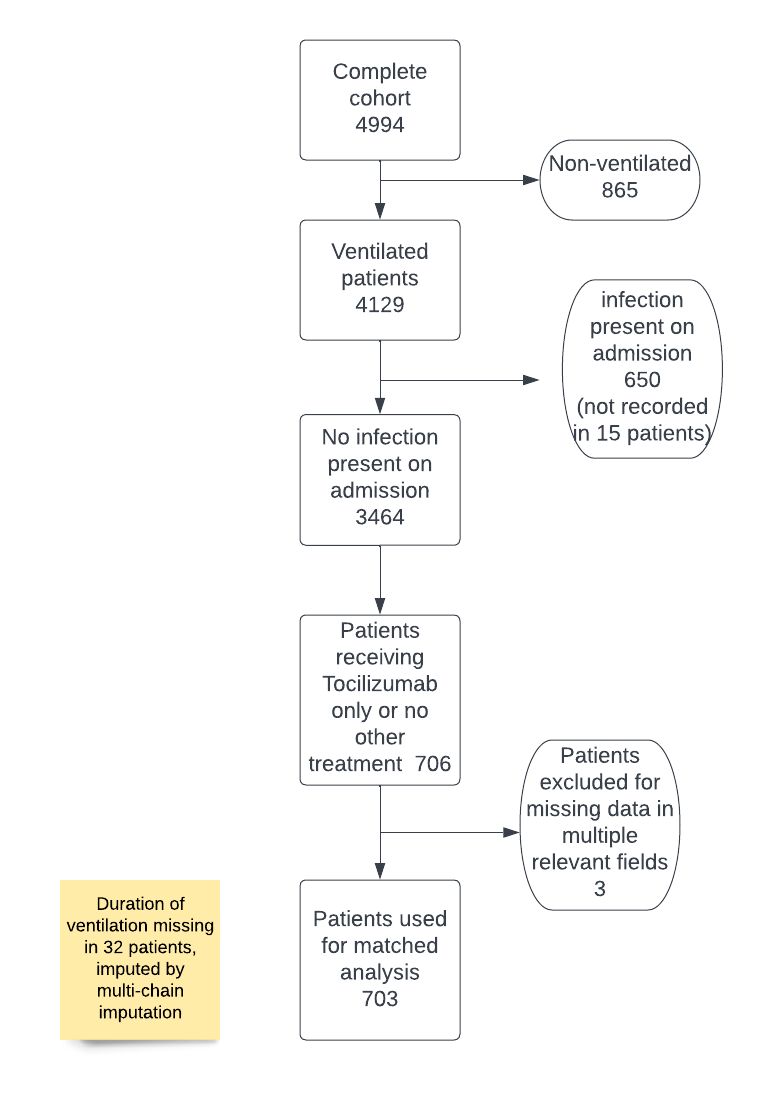


Figure S3: Propensity score (PS) matching for corticosteroid matching parameters compared to the input cohort. A density plots and histograms showing the effect of PS matching on distributions. B Covariance balance ‘love’ plot illustrating the effect of PS on standardised mean difference. C Numeric summary statistics following PS matching. Reporting percentages for categorical variables, mean for non-skewed parameters, median for skewed parameters. Last two entries below the thick line are outcome measures and were not used for matching.


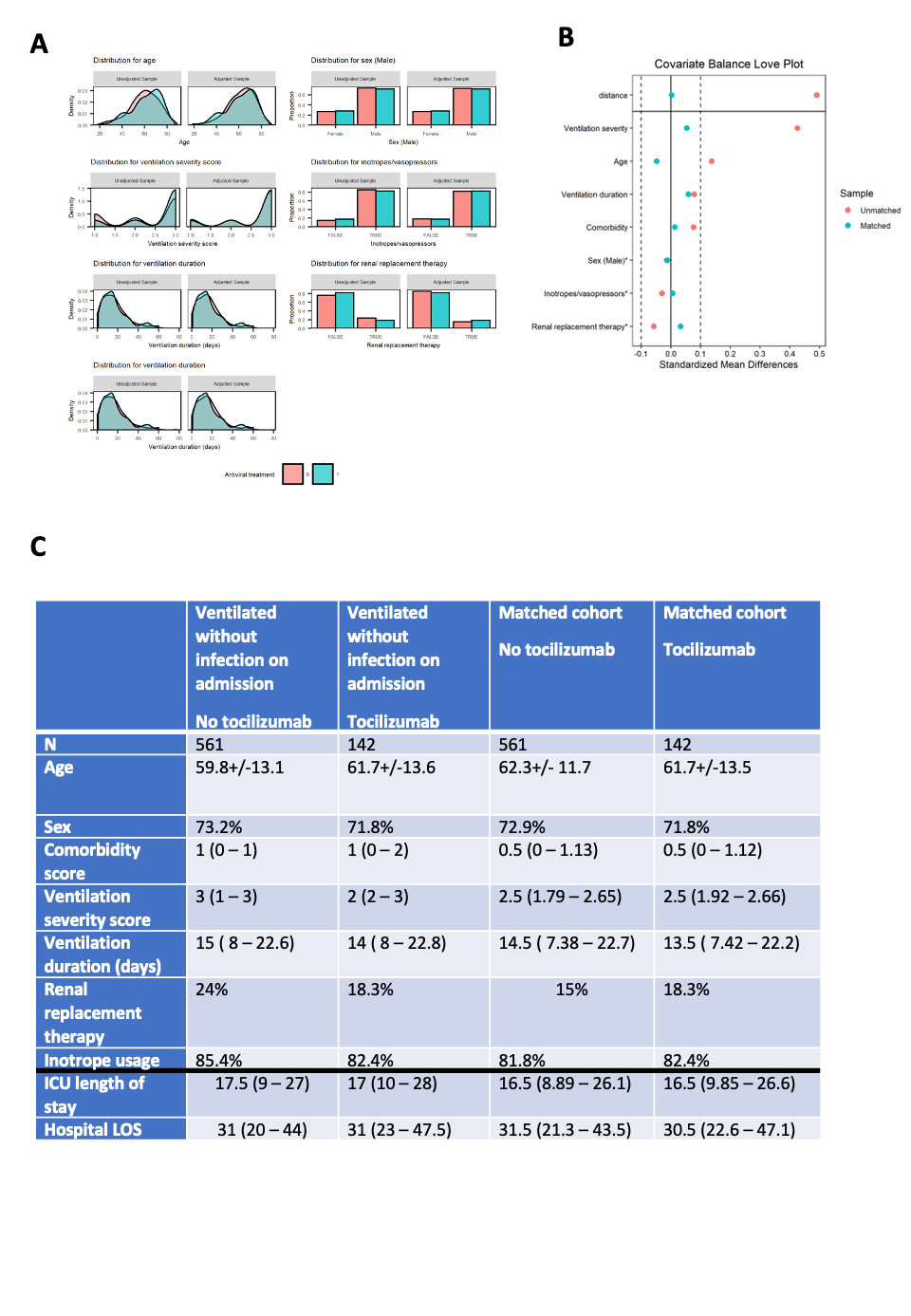


**The ESICM UNITE COVID study group members**

**ARGENTINA:** Hospital de Agudos Santojanni (Buenos Aires): Marco Bezzi; Hospital Universitario Austral (Buenos Aires): Alicia Gira;

**AUSTRIA:** Medical University of Graz (Graz): Philipp Eller;

**BANGLADESH:** Asgar Ali Hospital (Dhaka): Tarikul Hamid; Central Police Hospital (Dhaka): Injamam Ull Haque;

**BELGIUM:** AZ Rivierenland (Bornem): Wim De Buyser; CHIREC Hospitals (Brussels): Antonella Cudia, Daniel De Backer, Pierre Foulon ; Cliniques de l'Europe, St-Michel (Brussels): Vincent Collin; Universitair Ziekenhuis Gent (Gent): Jan De Waele, Jolien Van Hecke; UZ Brussel (Jette): Elisabeth De Waele, Claire Van Malderen; CH Jolimont (La Louvière): Jean-Baptiste Mesland; CHU Charleroi (Lodelinsart): Michael Piagnerelli; CHU Ambroise Pare (Mons): Lionel Haentjens; Clinique Saint-Pierre (Ottignies): Nicolas De Schryver; GZA Ziekenhuizen (Wilrijk): Jan Van Leemput, Philippe Vanhove; Mont-Godinne University Hospital, CHU UCL Namur (Yvoir): Pierre Bulpa;

**BULGARIA:** Multidisciplinary Hospital for Pulmonary Diseases St. Sofia (Sofia): Viktoria Ilieva;

**CANADA:** Brampton Civic Hospital (Brampton): David Katz; North York General Hospital (Toronto): Anna Geagea; William Osler Health System - Etobicoke General Hospital (Toronto): Alexandra Binnie;

**CHILE:** Complejo Asistencial Dr. Victor Rios Ruiz (Los Angeles): Fernando Tirapegui; Hospital Clínico Fusat (Rancagua): Gustavo Lago; Clínica Alemana de Santiago (Santiago): Jerónimo Graf, Rodrigo Perez-Araos; Hospital del Salvador (Santiago): Patricio Vargas; Facultad de Medicina, Escuela de Medicina, Universidad Andrés Bello (Viña del Mar): Felipe Martinez; Hospital Naval Almirante Nef (Viña del Mar): Eduardo Labarca;

**COLOMBIA**: Hospital San Jose (Bogota): Daniel Molano Franco; Universidad de La Sabana (Chía) and Clínica Universidad de La Sabana (Chía): Daniela Parra-Tanoux, Luis Felipe Reyes; Ces Clinic (Medellin): David Yepes;

**CROATIA:** University Hospital Split (Split): Filip Periš, Sanda Stojanović Stipić;

**ECUADOR**: Hospital General Guasmo Sur (Guayaquil): Cynthia Vanessa Campozano Burgos, Paulo Roberto Navas Boada; Hospital de Especialidades Portoviejo (Portoviejo): Jose Luis Barberan Brun, Juan Pablo Paredes Ballesteros;

**EGYPT**: Gamal Abdelnasser (Alexandria): Ahmed Hammouda; Wingat Royal Hospital (Alexandria): Omar Elmandouh; Luxor Pyretic Medical Centre (Armant): Ahmed Azzam; Assiut University Hospital (Assiut): Aliae Mohamed Hussein; Aswan University (Aswan): Islam Galal; Ain-Shams University Hospitals (Cairo): Ahmed K. Awad; Kasr Al Ainy Cairo University Hospital (Cairo): Mohammed A Azab; Misr International Hospital (Cairo): Maged Abdalla, Hebatallah Assal, Mostafa Alfishawy; El-Sheikh Zayed Specialized Hospital (Giza): Sherief Ghozy; Mansoura University Hospitals (Mansoura): Samar Tharwat; Elmenshawy General Hospital (Tanta): Abdullah Eldaly;

**ESTONIA**: Tartu University Hospital (Tartu): Veronika Reinhard;

**FRANCE**: Hôpital d’Instruction des Armées Percy (Clamart): Anne Chrisment, Chrystelle Poyat; Hôpital Nord Franche-Comté (Trevenans) : Julio Badie, Fernando Berdaguer Ferrari;

**GERMANY:** Charité - Universitätsmedizin Berlin, ICU 8i (Berlin): Björn Weiss; Charité - Universitätsmedizin Berlin, ICU 43i (Berlin): Karl Friedrich Kuhn; Charité - Universitätsmedizin Berlin, ICU 44i (Berlin): Julius J. Grunow; Charité - Universitätsmedizin Berlin, ICU 144i (Berlin): Marco Lorenz; Charité - Universitätsmedizin Berlin, 203i (Berlin): Stefan Schaller; University Hospital Dresden (Dresden): Peter Spieth; Bethesda Krankenhaus Bergedorf (Hamburg): Marc Bota; University Hospital Leipzig (Leipzig): Falk Fichtner; Klinikum Rechts der Isar der TUM, IS1/M2B (Munich): Kristina Fuest; Klinikum Rechts der Isar der TUM, R3A (Munich): Tobias Lahmer; University Hospital of Wurzburg (Wurzburg): Johannes Herrmann, Patrick Meybohm;

**GREECE:** General Hospital of Eleusis ‘Thriasion’ (Eleusis): Nikolaos Markou; George Papanikolaou General Hospital (Exohi-Thessaloniki): Georgia Vasileiadou; University Hospital Attikon: (Haidari): Evangelia Chrysanthopoulou; General Hospital of Larissa (Larissa): Panagiotis Papamichalis; University General Hospital of Thessaloniki AHEPA (Thessaloniki): Ioanna Soultati;

**INDIA**: Deenanath Mangeshkar Hospital and Research Center (Pune): Sameer Jog; Tata Memorial Hospital, Homi Bhabha National University (Mumbai) : Kushal Kalvit; Sheila Nainan Myatra;

**IRELAND**: Cavan General Hospital (Cavan): Ivan Krupa; Our Lady of Lourdes Hospital (Drogheda): Aisa Tharwat; St Vincent's University Hospital (Dublin): Alistair Nichol; Galway University Hospitals (Galway): Aine McCarthy;

**IRAN**: Imam Reza Hospital (Tabriz): Ata Mahmoodpoor;

**ITALY**: Sant'Orsola University Hospital (Bologna): Tommaso Tonetti; Santissima Trinità Hospital (Cagliari): Paolo Isoni; Arcispedale Sant’Anna (Ferrara): Savino Spadaro, Carlo Alberto Volta; University of Foggia Ospedali riuniti Foggia (Foggia): Lucia Mirabella; AOU G. Martino (Messina): Alberto Noto; Fondazione IRCCS Ca' Granda Ospedale Maggiore Policlinico (Milan): Gaetano Florio, Amedeo Guzzardella, Chiara Paleari; IRCCS Humanitas Research Hospital (Milan): Federica Baccanelli, Marzia Savi; Gemelli IRCCS (Rome): Massimo Antonelli; San Luca (Trecenta Rovigo): Barbara Vaccarini; Città della Salute e della Scienza - Presidio Molinette (Turin): Giorgia Montrucchio, Gabriele Sales; University Hospital Integrated Trust (AOUI) Of Verona (Verona): Katia Donadello, Leonardo Gottin, Enrico Polati; San Bortolo Hospital (Vicenza): Silvia De Rosa;

**KENYA**: MP Shah Hospital (Nairobi): Demet Sulemanji;

**LIBYA**: Almwasfat Hospital (Tripoli): Abdurraouf Abusalama; Tripoli University Hospital (Tripoli): Muhammed Elhadi;

**MEXICO**: Hospital General De Ecatepec Las Americas (Ecatepec de Morelos): Montelongo Felipe De Jesus; Hospital civil nuevo dr Juan I Menchaca (Guadalajara Jalisco): Daniel Rodriguez Gonzalez; Hospital de Especialidades Dr.Antonio Fraga Mouret CMN La Raza (Mexico): Nancy Canedo, Alejandro Esquivel Chavez;

**MOROCCO**: Ibn Sina University Hospital (Rabat): Tarek Dendane;

**NETHERLANDS**: Ziekenhuisgroep Twente(Almelo): Bart Grady, Ben de Jong; Amsterdam UMC, VUmc site (Amsterdam): Eveline van der Heiden, Patrick Thoral; Onze Lieve Vrouwe Gasthuis (Amsterdam): Bas van den Bogaard; Gelre Ziekenhuizen (Apeldoorn): Peter E. Spronk; Haaglanden Medisch Centrum (Den Haag): Sefanja Achterberg; Deventer Ziekenhuis (Deventer): Melanie Groeneveld; Albert Schweitzer Hospital (Dordrecht): Ralph K.L. So, Calvin de Wijs; Catharina Ziekenhuis (Eindhoven): Harm Scholten; Medisch Spectrum Twente (Enschede): Albertus Beishuizen, Alexander D. Cornet; Martiniziekenhuis (Groningen): Auke C. Reidinga; University Medical Center Groningen (Groningen): Hetty Kranen, Roos Mensink; Spaarne Gasthuis (Haarlem): Sylvia den Boer, Marcel de Groot; Tjongerschans Heerenveen (Heerenveen): Oliver Beck; Medical Centre Leeuwarden (Leeuwarden): Carina Bethlehem; Maastricht University Medical Center (Maastricht): Bas van Bussel; Radboudumc (Nijmegen): Tim Frenzel; Elisabeth TweeSteden Ziekenhuis (ETZ) (Tilburg): Celestine de Jong, Rob Wilting; University Medical Center Utrecht (Utrecht): Jozef Kesecioglu; VieCuri Medical Center (Venlo): Jannet Mehagnoul-Schipper;

**NIGERIA**: University of Port Harcourt Teaching Hospital (Port Harcourt): Datonye Alasia;

**PAKISTAN**: Ziauddin Hospital Clifton Campus (Karachi): Ashok Kumar; Bahria International Hospital (Lahore): Ahad Qayyum, Muhammad Rana;

**PALESTINE**: Alshifaa Hospital (Gaza) : Mustafa Abu Jayyab;

**PERU**: Hospital Nacional Dos de Mayo (Lima): Rosario Quispe Sierra;

**PHILIPINES**: Asian Hospital and Medical Center (Muntinlupa): Aaron Mark Hernandez;

**PORTUGAL**: Hospital de Cascais - Dr. José de Almeida (Alcabideche): Lúcia Taborda; Hospital Prof. Dr. Fernando da Fonseca, E.P.E. (Amadora): Tiago Ramires; Centro Hospitalar e Universitário de Coimbra (Coimbra): Catarina Silva; Centro Hospitalar de Leiria (Leiria): Carolina Roriz; Hospital São Francisco Xavier (Lisboa): Pedro Póvoa; Hospital Beatriz Ângelo (Loures): Patricia Patricio; Centro Hospitalar e Universitário São João, Infectious Diseases Intensive Care Unit (Porto): Maria Lurdes Santos; Centro Hospitalar Universitário de São João, Serviço de Medicina Intensiva (Porto): Vasco Costa, Pedro Cunha; Centro Hospitalar Universitário do Porto, Hospital Santo Antonio (Porto): Celina Gonçalves; Centro Hospitalar de Entre o Douro e Vouga (Santa Maria da Feira): Sandra Nunes; Hospital Pedro Hispano (Senhora da Hora): João Camões; Centro Hospitalar Vila Nova de Gaia/Espinho (Vila Nova de Gaia): Diana Adrião; Centro Hospitalar de Tondela-Viseu, EPE (Viseu): Ana Oliveira;

**QATAR**: **Hamad Medical Corporation (Doha)**: Alwakra Hospital (Alwakra): Ali Omrani; Hamad General Hospital, HGH ICU (Doha): Muna Al Maslamani; Hamad General Hospital, HMC- MICU (Doha): Abdurrahmaan Suei elbuzidi; Hamad Medical Corporation, Accident and Emergency (Doha): Bara Mahmoud Al qudah; Hazem Mubarak General Hospital, HMGH-1 (Doha): Abdel Rauof Akkari, Mohamed Alkhatteb; Hazem Mubarak General Hospital, HMGH-2 (Doha): Anas Baiou; Hazem Mubarak General Hospital, HMGH-3: Ahmed Husain; Hazem Mubarak General Hospital, HMGH-4 (Doha): Mohamed Alwraidat, Ibrahim Abdulsalam Saif; Hazem Mubarak General Hospital, HMGH-5 (Doha): Dana Bakdach; Hazem Mubarak General Hospital, HMGH-6 (Doha): Amna Ahmed, Mohamed Aleef; The Cuban Hospital, TCH ICU (Dukhan): Awadh Bintaher;

**ROMANIA**: Clinical Emergency County Hospital (Cluj-Napoca): Cristina Petrisor;

**RUSSIA**: State budgetary healthcare institution ‘Research Institute-regional clinical hospital named after Professor Ochapovsky S.V’ (Krasnodar): Evgeniy Popov; City Clinical Hospital № 40 (Moscow): Ksenia Popova; Federal State Budgetary Institution ‘National Medical and Surgical Center named after N.I. Pirogov’ of the Ministry of Healthcare of the Russian Federation (Moscow): Mariia Dementienko; FGBU ‘National Medical-Surgery Hospital by N.I.Pirogov’ (Moscow): Boris Teplykh; FSBI <National Medical Research Center for Obstetrics, Gynaecology and Perinatology named after Academician V.I. Kulakov> Ministry of Healthcare of the Russian Federation (Moscow): Alexey Pyregov; Moscow City Hospital N. 52 (Moscow): Liubov Davydova; Privolzhskiy District Medical Center (Nizhny Novgorod): Belskii Vladislav; Novosibirsk State University with clinical facility City Clinical Hospital #25 (Novosibirsk): Elena Neporada, Ivan Zverev; Botkin's Hospital (St. Petersburg): Svetlana Meshchaninova; First Pavlov State Medical University of St. Petersburg, Anesthesiology and Intensive Care №2 (St. Petersburg): Dmitry Sokolov; First Pavlov State Medical University of St. Petersburg, ICU №2 (St. Petersburg): Elena Gavrilova; First Pavlov State Medical University of St. Petersburg, Scientific Clinical Center of Anesthesiology and Resuscitation (St. Petersburg): Irena Shlyk; Saint Petersburg State Medical Institution ‘City Hospital No. 38 named after N. A. Semashko’ (St. Petersburg): Igor Poliakov; War Veteran’s Hospital, СПб ГБУЗ Госпиталь для ветеранов войн (St. Petersburg): Марина Власова;

**SAUDI ARABIA**: Pharmacy Practice Department, Faculty of Pharmacy, King Abdulaziz University Hospital (Jeddah): Ohoud Aljuhani, Amina Alkhalaf; King Abdulaziz Medical City (Riyadh): Felwa Bin Humaid, Yaseen Arabi; King Saud Medical City: Ahmed Kuhail; Prince Sultan Medical Military Center, GICU1 (Riyadh): Omar Elrabi; Prince Sultan Medical Military Center, GICU2 (Riyadh): Madihah Alghnam;

**SINGAPORE**: Ng Teng Fong General Hospital, Jurong Health, NUHS (Singapore): Amit Kansal; Sengkang General Hospital (Singapore): Vui Kian Ho; Tan Tock Seng Hospital (Singapore): Jensen Ng;

**SPAIN**: Complejo Hospitalario Universitario de A Coruña (A Coruña): Raquel Rodrígez García, Xiana Taboada Fraga; Hospital General La Mancha Centro (Alcázar de San Juan): Mª del Pilar García-Bonillo, Antonio Padilla-Serrano; Hospital Universitario San Agustín (Aviles): Marta Martin Cuadrado; Hospital Clinic Barcelona (Barcelona): Carlos Ferrando; Hospital General Universitario de Castellon (Castellon de la Plana): Ignacio Catalan-Monzon, Laura Galarza; Hospital Universitario de Getafe (Getafe): Fernando Frutos-Vivar, Jorge Jimenez, Carmen Rodríguez-Solis; Hospital San Jorge (Huesca): Enric Franquesa-Gonzalez; Complejo Hospitalario Insular Materno Infantil (Las Palmas de Gran Canaria): Guillermo Pérez Acosta, Luciano Santana Cabrera; Hospital Universitario Severo Ochoa (Leganes): Juan Pablo Aviles Parra, Francisco Muñoyerro Gonzalez; Hospital Rafael Mendez (Lorca): Maria del Carmen Lorente Conesa; Hospital Universitario Lucus Augusti (Lugo): Ignacio Yago Martinez Varela; Hospital HM Sanchinarro (Madrid): Orville Victoriano Baez Pravia; Hospital Universitario de Torrejón (Madrid): Maria Cruz Martin Delgado, Carlos Munoz de Cabo; Hospital Universitario Fundacion Jimenez Diaz (Madrid): Ana-Maria Ioan, Cesar Perez-Calvo, Arnoldo Santos; Hospital Universitario Infanta Leonor (Madrid): Ane Abad-Motos, Javier Ripolles-Melchor; Hospital Universitario La Paz (Madrid): Belén Civantos Martin, Santiago Yus Teruel; Hospital Universitario Príncipe de Asturias (Madrid): Juan Higuera Lucas; Hospital Universitario Ramón y Cajal (Madrid): Aaron Blandino Ortiz, Raúl de Pablo Sánchez; Regional University Hospital of Malaga (Malaga): Jesús Emilio Barrueco-Francioni; Hospital Universitario Central de Asturias (Oviedo): Lorena Forcelledo Espina; Hospital Quironsalud Palmaplanas (Palma de Mallorca): José M. Bonell-Goytisolo; H.U. Son Llàtzer (Palma de Mallorca): Iñigo Salaverria, Antonia Socias Mir; Complejo Hospitalario Universitario de Santiago de Compostela (Santiago de Compostela): Emilio Rodriguez-Ruiz; Complejo Asistencial de Segovia (Segovia): Virginia Hidalgo Valverde, Patricia Jimeno Cubero; Hospital Nuestra Señora Del Prado (Talavera de la Reina): Francisca Arbol Linde, Nieves Cruza Leganes; Hospital Provincial de Toledo (Toledo): Juan Maria Romeu; Hospital Verge de la Cinta (Tortosa): Pablo Concha; Hospital Universitario Río Hortega, Servicio de Medicina Intensiva (Valladolid): José Angel Berezo-Garcia, Virginia Fraile; Hospital Universitario Río Hortega, Servicio de Medicina Intensiva, Unidad 2 (Valladolid): Cristina Cuenca-Rubio, David Perez-Torres; Hospital Clínic Universitari de Valencia (Valencia): Ainhoa Serrano; Hospital Universitario de La Plana (Vila-Real): Clara Martínez Valero; Hospital Comarcal Vinaroz (Vinaroz): Andrea Ortiz Suner; Hospital Universitario de Alava (Vitoria-Gasteiz): Leire Larrañaga, Noemi Legaristi; Hospital Virgen de la Concha (Zamora): Gerardo Ferrigno;

**SUDAN:** Aliaa Specialist Hospital (Omdurman): Safa Khlafalla;

**SURINAME:** Academisch Ziekenhuis Paramaribo (Paramaribo): Rosita Bihariesingh-Sanchit;

**SWEDEN**: Hallands Sjukhus (Halmstad): Frank Zoerner; Karolinska University Hospital (Huddinge): Jonathan Grip, Kristina Kilsand; Sunderby Hospital (Luleå): Jonas Österlind; Akademiska Sjukhuset, Uppsala Univeristy Hospital (Uppsala): Magnus von Seth; Västerviks Sjukhus (Västerviks): Johan Berkius;

**SWITZERLAND**: Clinica Luganese Moncucco (Lugano): Samuele Ceruti, Andrea Glotta;

**TURKEY**: Ankara City Hospital / General Hospital (Ankara): Seval Izdes; Ankara City Hospital Orthopedics and Neurology Hospital (Ankara): Işıl Özkoçak Turan; Gulhane Egitim ve Arastirma Hastanesi (Ankara): Ahmet Cosar; Hacettepe University (Ankara): Burcin Halacli; University of Health Sciences Kecioren Training and Research Hospital (Ankara): Necla Dereli; Derince Research and Education Hospital, Health Sciences University (Derince / Kocaeli): Mehmet Yilmaz; Düzce University School of Medicine (Düzce): Türkay Akbas; Gaziantep University (Gaziantep): Gülseren Elay; Giresun Üniversitesi Prof. Dr. A. İlhan Özdemir Eğitim Araştırma Hastanesi (Giresun): Selin Eyüpoğlu; Kartal Dr. Lütfí Kirdar Şehír Hastanesí (Istanbul): Yelíz Bílír, Kemal Tolga Saraçoğlu; SBU Kanuni Sultan Suleyman Education and Training Hospital (Istanbul): Ebru Kaya, Ayca Sultan Sahin; Ege University School of Medicine (Izmir): Pervin Korkmaz Ekren; Niğde Research and Training Hospital (Niğde): Tuğçe Mengi; Sakarya University Education Research Hospital (Sakarya): Kezban Ozmen Suner, Yakup Tomak; Kanuni Education and Training Hospital (Trabzon): Ahmet Eroglu;

**UNITED ARAB EMIRATES:** Mediclinic City Hospital (Dubai): Asad Alsabbah;

**UNITED KINGDOM:** Aberdeen Royal Infirmary (Aberdeen): Katie Hanlon; Belfast City Hospital (Belfast): Kevin Gervin, Sean McMahon; Ulster Hospital (Belfast): Samantha Hagan; Queen Elizabeth Hospital, University Hospitals Birmingham NHS Foundation Trust (Birmingham): Caroline V Higenbottam, Randeep Mullhi, Lottie Poulton, Tomasz Torlinski; Royal Blackburn Hospital (Blackburn): Allen Gareth, Nick Truman; West Suffolk Hospital NHS foundation Trust (Bury St Edmunds): Gopal Vijayakumar; Cambridge University Hospitals (Cambridge): Chris Hall, Alasdair Jubb; Royal Papworth Hospital NHS Foundation Trust (Cambridge): Lenka Cagova, Nicola Jones; Countess Of Chester (Chester): Sam Graham, Nicole Robin; Darlington Memorial Hospital (Darlington): Amanda Cowton; Altnagelvin Hospital - WHSCT (Derry): Adrian Donnelly; Doncaster Royal Infirmary (Doncaster): Natalia Singatullina; University Hospital of North Durham (Durham): Melanie Kent; Royal Devon & Exeter NHS Foundation Trust (Exeter): Carole Boulanger; Royal Surrey Hospital (Guildford): Zoë Campbell, Elizabeth Potter; Royal Gwent Hospital (Gwent): Natalie Duric, Tamas Szakmany; Harefield Hospital, Royal Brompton and Harefield NHS Foundation Trust (Harefield): Orinta Kviatkovske, Nandor Marczin; The Princess Alexandra Hospital NHS Trust (Harlow): Caroline Ellis, Rajnish Saha; Harrogate District Hospital (Harrogate): Chunda Sri-Chandana; NHS University Hospital Crosshouse (Kilmarnock): John Allan; Kingston Hospital (Kingston upon Thames): Lana Mumelj, Harish Venkatesh; University Hospitals of Morecambe Bay NHS Foundation Trust, Royal Lancaster Infirmary (Lancaster): Vera Nina Gotz; St Helens and Knowsley Teaching Hospitals NHS Trust (Liverpool): Anthony Cochrane; Guy's & St Thomas' Hospital (London): Nuttha Lumlertgul, Barbara Ficial; Homerton University Hospital NHS Foundation Trust (London): Susan Jain; Royal Brompton Hospital, Royal Brompton and Harefield NHS Foundation Trust (London): Giulia Beatrice Crapelli, Aikaterini Vlachou; Maidstone Hospital (Maidstone): David Golden; Borders General Hospital (Melrose): Sweyn Garrioch; James Cook University (Middlesbrough): Jeremy Henning, Gupta Loveleena; The Tunbridge Wells Hospital (Pembury): Miriam Davey; Queen's Hospital (Romford): Lina Grauslyte, Erika Salciute-Simene; Salisbury NHS Foundation Trust (Salisbury): Martin Cook; Stepping Hill Hospital (Stockport): Danny Barling, Phil Broadhurst; University Hospital of North Tees (Stockton-on-Tees): Sarah Purvis; Royal Cornwall Hospitals NHS Trust (Truro): Spivey Michael; Warwick Hospital (Warwick): Benjamin Shuker; Royal Hampshire County Hospital (Winchester): Irina Grecu; Queen Elizabeth Hospital (Woolwich): Daniel Harding; Bassetlaw District General Hospital (Worksop): Natalia Singatullina;

**UNITED STATES**: University of New Mexico School of Medicine (Albuquerque, NM): James T. Dean, Nathan D. Nielsen; Brooklyn VA Medical Center (Brooklyn, VA): Sama Al-Bayati; SUNY Downstate Medical Center (Brooklyn, NY): Mohammed Al-Sadawi; Cooper University Hospital (Camden, NJ): Mariane Charron; St. Joseph Hospital (Denver, CO): Peter Stubenrauch; Ochsner Medical Center (New Orleans, LA): Jairo Santanilla, Catherine Wentowski; University of Utah Health (Salt Lake City, UT): Dorothea Rosenberger; Stony Brook University Hospital (Stony Brook, NY): Polikseni Eksarko, Randeep Jawa;
